# Immuno-acoustic trapping for extracellular vesicle subpopulations

**DOI:** 10.1038/s41598-025-33842-6

**Published:** 2025-12-31

**Authors:** Axel Broman, Megan Havers, Roman Sattarov, Thomas Laurell

**Affiliations:** 1 Department of Biomedical Engineering, Lund University, Lund, Sweden; 2 Department of Clinical Sciences, Lund University, Lund, Sweden

**Keywords:** Immuno-acoustic trapping, Extracellular vesicles, Acoustic trapping, Subpopulation, Immunoaffinity, Proteomics, Biological techniques, Biotechnology, Immunology

## Abstract

Extracellular vesicles (EVs) in biofluids are heterogeneous in origin, surface protein expression, and biomolecular cargo. Isolation of specific EV subpopulations may enable studies of biomarkers originating from specific cell phenotypes and disease states. Acoustic trapping is a rapid, automatable EV isolation technique requiring minimal sample preprocessing; however, it lacks the specificity needed for some clinical studies. Combining acoustic trapping with immunoaffinity bead-based isolation could provide access to more specific EV content and broaden the applications of acoustic trapping. Here, we present a novel immuno-acoustic trapping methodology implemented on an automated platform with simple design. EVs were rapidly isolated (8 min) from ~ 17 µL blood plasma, generating two EV fractions from a single trapping run: acoustically trapped EVs and EVs bound specifically to anti-CD9 silica beads. We benchmark our technique against a manual (90 min) immunoaffinity isolation with multiple centrifugation washes. Nanoparticle tracking analysis and transmission electron microscopy with CD9^+^ immunogold labeling confirmed isolation of intact EVs. Quantitative proteomic profiling showed that immuno-acoustic fractions contained proteins more strongly associated with CD9 than both acoustically and immunoaffinity-isolated fractions. This methodology can be adapted to isolate specific EV subpopulations expressing other surface proteins while simultaneously collecting a broad EV population as background reference.

**Supplementary Information:**

The online version contains supplementary material available at 10.1038/s41598-025-33842-6.

## Introduction

Extracellular vesicles (EVs) are biological nanoparticles enclosed by lipid bilayer membranes, they are secreted from cells and mediate intercellular communication. The cell of origin and mode of biogenesis affect the composition of EVs. EVs are heterogeneous, typically containing many proteins, nucleic acids, lipids and metabolites. The tetraspanins CD9, CD81 and CD63 are widely used markers of EV subsets and have been used as targets to immuno-isolate EVs. CD9, a protein with four transmembrane domains, is expressed on the plasma membrane and is highly abundant on EVs^1,2^.

EVs are usually either isolated by immunoaffinity or physical properties. Immunoaffinity-based techniques use antibodies or ligands to target specific particles possessing a membrane protein^3^, usually captured with functionalized beads^4^. Ultracentrifugation and acoustic trapping are techniques relying on the physical characteristic of EVs being denser than the media they are in, isolating nanoparticles by centrifugal or acoustic forces, respectively. Ultracentrifugation is typically performed with multiple steps, over several hours and forces in the range of 100,000x *g* to pellet small EVs. Acoustic trapping is a rapid, label-free, automated system whereby seed particles levitated in a localized acoustic field enable enrichment of submicron particles^5^. EV enrichment and washing from small volumes has been reported for blood plasma, urine, cerebrospinal fluid and cell media^6–11^.

The mechanism of acoustic trapping of microparticles and cells is primarily driven by a difference in density between the particles and the fluid that they are in. When an acoustic standing wave is generated by applying ultrasound at the resonant frequency of a fluid-filled glass capillary, particles that are denser than the fluid will migrate to the pressure node of the acoustic field through acoustic radiation forces^12^. In an acoustic trapping capillary, the ultrasound transducer is small and localizes the acoustic field to the trapping region, this generates an energy density gradient in the flow direction which can retain particles against the flow^13^. Sub-micron particles such as extracellular vesicles cannot be directly trapped in such a field. However, when nanoparticles are close to larger sound-scattering seed particles (such as the silica particles of 10 μm used in this work), they experience interparticle forces and hydrodynamic effects which allow these nanoparticles to be trapped in the interstitial spaces^5^. This allows for washing of particles by switching buffer while the particles are retained in the trap against flow. After washing, the isolated EVs can be released by turning off the ultrasound and dispensing in a small volume of buffer.

Once isolated from background components, EVs can be scrutinized for their proteomic, lipidomic or transcriptomic profiles, as well as other molecular cargo. Although bulk population profiling has shown clinical value, to understand disease mechanisms and cell-specific EV content, a further refined grouping in multiple EV subpopulations could contribute additional diagnostic value. Immunoaffinity-isolation of EVs has already made great progress in isolating and identifying contents of EVs with surface proteins such as tetraspanins^14–16^ and/or for specific cell-types^17^. This idea has recently been capitalized on in research investigating the role of EVs in cancers^18^, neurodegeneration^19^, and fatty liver disease^17,20^, among other conditions. Isolation via immunoaffinity can enhance specificity and sensitivity in identifying low abundant biomarkers, for example by reducing the background of irrelevant EVs and solute proteins. Technological advances in microfluidics have created small devices for liquid handling, including the addition of mixing^21^, acoustic^22^ and magnetic^23^ manipulation. These are not used prevalently, in part due to the complicated device design and fabrication but also because low flowrates mean even small sample volumes can take hours to process.

**Fig. 1:**
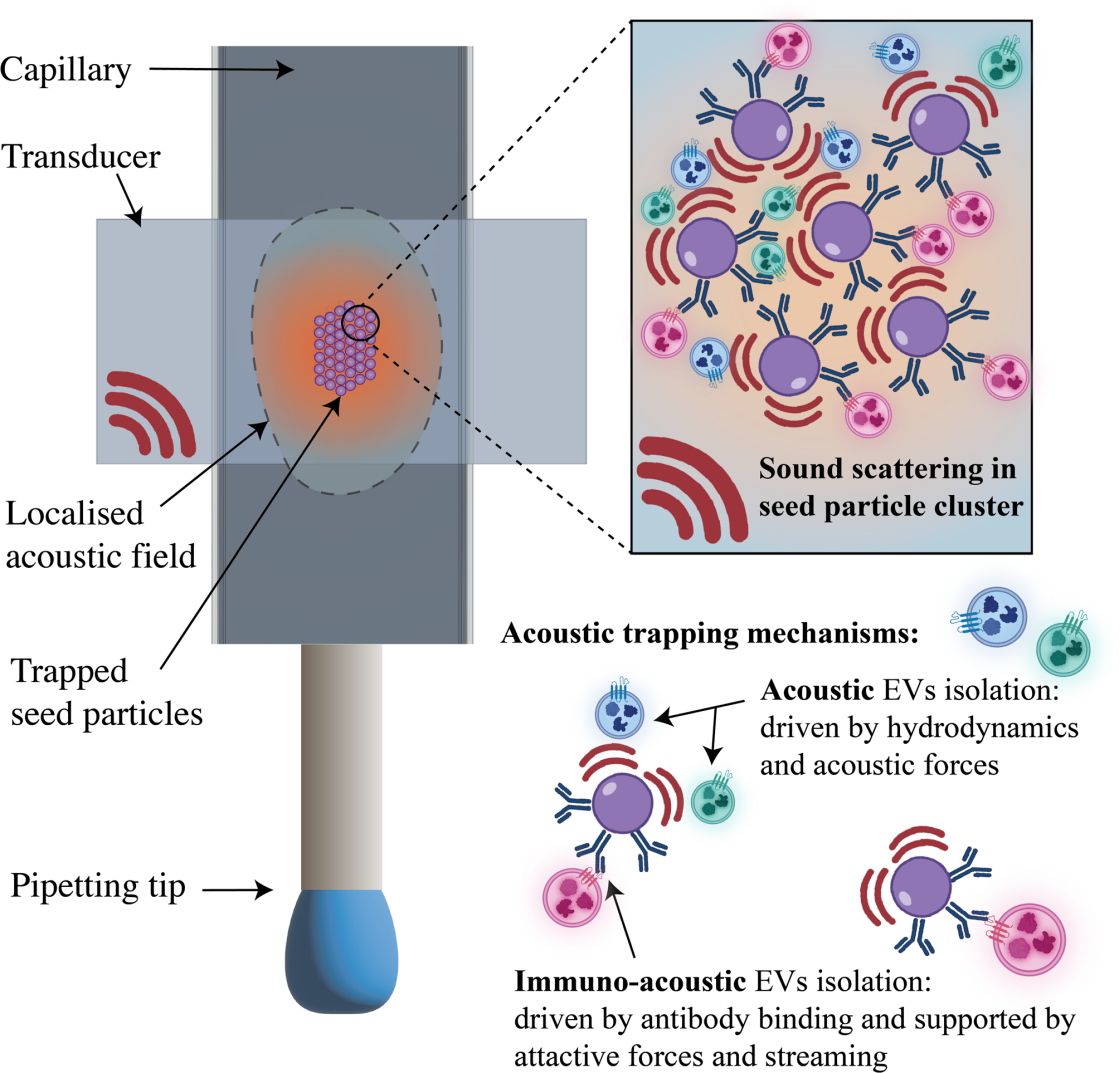
Schematic of acoustic trapping mechanisms taking place in our combined approach. The microfluidic capillary is vibrated with an ultrasound transducer at its resonant frequency, generating a localized acoustic field (illustrated with an orange glow). Traditional acoustic trapping is enabled by acoustically trapped microparticles (purple) which scatter sound (indicated in red) and experience radiation forces in the acoustic field, resulting in a cluster of these seed particles trapped in the pressure node. The resulting acoustic forces and hydrodynamic interactions enable EVs to be trapped between the seed particles, regardless of their surface marker expression. Immuno-acoustic trapping takes place at the same time as the acoustic trapping and is driven by the anti-CD9 binding (indicated in blue on the surface of the microparticles) to the antigens expressed on the CD9^+^ EVs.

In choosing an existing EV isolation technique, there is an inherent compromise between the sub-population bias of immunoaffinity, and the limited biological information deciphered from a non-specific EV population. Herein, we present a combined approach using immuno-acoustic isolation, demonstrating parallel isolation of two EV populations, Fig. 1. Using acoustic trapping with anti-CD9 functionalized silica seed particles, we have collected plasma EVs trapped by acoustic means whilst simultaneously isolating an EV subset by immunoaffinity binding of EVs to the seed particles in the acoustic trapping platform. The two sub-populations (acoustic and immuno-acoustic), once released from the trap, can be easily separated due to the fast sedimentation of the EV-bound silica seed particles while the acoustically trapped particles remain in suspension. In this investigation, we compare the EV size distribution, CD9 expression and protein cargo of the acoustically and immuno-acoustically isolated EVs. We also compare these to references samples of raw plasma and EVs isolated via immunoaffinity in a manual incubation method.

## Methods

### Platelet-poor blood preparation

The work was carried out in accordance with Helsinki declaration and following a protocol approved by the Swedish Ethical Review Authority (Ref. No. 2020-05818). Fresh blood was drawn from an anonymized healthy donor (after providing signed informed consent at the Biomedical Centre, Lund University, Sweden) into ethylenediamine tetraacetic acid (EDTA) vacutainer tubes (BD Bioscience, Temse, Belgium). 5 mL platelet poor plasma was obtained by centrifugating twice at 1500× *g* for 15 min and taking the supernatant. Plasma samples were processed fresh for the samples to be run by mass spectrometry (MS), for both manual and automated EV isolation methods. The remaining plasma was stored in 1 mL aliquots at − 80 °C and thawed before preparing EV samples for characterisation by nanoparticle tracking analysis (NTA) and transmission electron microscopy (TEM).

### Functionalized silica seed particles

10 μm silica microparticles with protein-A coating (Lot #GK0332343-01, Kisker Biotech) were incubated with CD9 Recombinant Rabbit Monoclonal Antibody (SA35-08) (Catalogue # MA5-31980, Thermofisher) for 1 h, then washed twice by centrifugation of beads at 200× *g* for 1 min. To reduce the risk of dissociation, we used the seed particles the same day as functionalization. The temperature inside the trap remained in the range of 25–35 °C during the trapping process. For verification of the method, this process was also performed with a fluorescently labelled antibody: BD™ PE Mouse Anti-Human CD9 (Catalogue #341647, BD Biosciences). The PE-antiCD9-proteinA-Silica particles were quantified by flow cytometry (FACS Canto II, BD biosciences) using the R-Phycoerythrin (PE) fluorescence excitation 488 nm, filter 585/42. Unlabeled proteinA-Silica particles were used set the gate by PE fluorescence intensity, before quantifying the percentage of PE-antiCD9-proteinA-Silica particles which were PE positive.

### Simultaneous acoustic and immuno-acoustic isolation

**Fig. 2:**
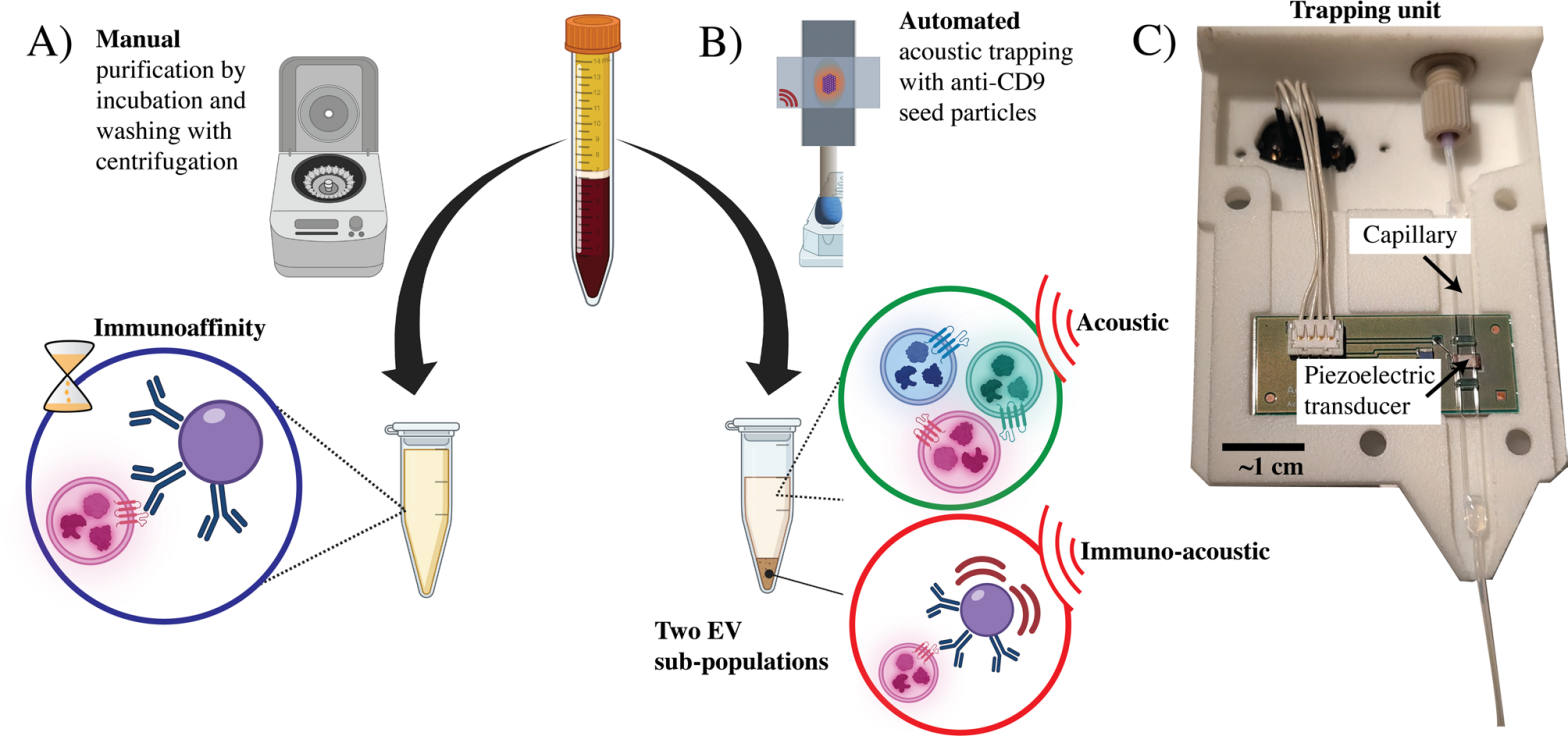
Sample processing schematic of the parallel CD9^+^ EV isolation methods, manual and automated. Both methods make use of anti-CD9 functionalized beads and in each protocol 50 µL of platelet-poor plasma diluted in PBS (1:2) was processed. (**A**) In the manual method (left arrow) the plasma is incubated for 90 min with the beads for immunoaffinity capture, followed by 3 washing steps (centrifugation and pipetting), before resuspending the EVs which are positive for CD9 and are bound to the beads. (**B**) In the automated method, plasma EVs are acoustically trapped using the antibody-functionalized beads as seed particles and washed rapidly. After resuspending and pelleting the beads—the supernatant contains trapped EVs not chemically bound to the seed particles and the pellet contains the sub-population of EVs isolated which were positive for CD9. (**C**) Photograph of the trapping unit. Figure created with Biorender.com.

Acoustic trapping was performed on the AcouTrap 2 system (AcouSort AB, Sweden), with a single node capillary actuated at 4.1 MHz, as previously described^24^. AntiCD9-proteinA-silica seed particles were preloaded, a full cluster in our capillary contains around 60,000 silica 10 μm beads^24^. Then fresh plasma (50 µL of 1:2 plasma: PBS) was aspirated, with 6 technical replicates. Following washing with 200 µL at a flowrate of 200 µL/min, the seed particle cluster was eluted in 75 µL of PBS at 2000 µL/min. After agitation by vortexing, the sample was fractionated by centrifugation at 200× *g* for 1 min. The supernatant (65 µL) was taken as the acoustically trapped EV fraction, Fig. 2. The pellet was further washed twice in 400 µL PBS and resuspended in 70 µL. The immuno-acoustically trapped EVs were released from the beads with 5 µL formic acid (10%) and collected in the supernatant following centrifugation at 200× *g* for 1 min.

### Immunoaffinity isolation

As a reference, plasma samples were processed in parallel by the manual immunoaffinity isolation method, as indicated in Fig. 2. The same volume of antiCD9-proteinA-silica particles was used to isolate CD9^+^ EVs from plasma (50 µL of 1:2 plasma: PBS), with 6 technical replicates. After 90 min incubation on a tube rotator at room temperature, the immunoaffinity captured EVs were washed to remove free protein and CD9^−^ EVs. The pellet was washed with 400 µL PBS (centrifugation 200× *g* for 1 min) three times and finally resuspended in 70 µL. The EVs were released with 5 µL formic acid (10%) and collected in the supernatant following centrifugation at 200× *g* for 1 min.

### Nanoparticle tracking analysis (NTA)

Nanoparticle tracking analysis (NTA, LM10, Malvern Analytical) was used to quantify the size and approximate concentration of the isolated EVs. Diluted plasma, manually isolated EVs and both fractions of automatically isolated EVs were measured at camera level 15. For each sample, 3 videos were recorded of 90 s duration and analysed with detection threshold 3.

### Transmission electron microscopy (TEM)

The CD9 presence on EVs in raw plasma and each of the isolated EV populations was investigated by transmission electron microscopy (TEM, FEI Tecnai Biotwin 120 kV), using 1 technical replicate. Before imaging, samples were deposited on grids and either labelled with both anti-CD9 and a colloidal gold (10/15 nm) goat anti-rabbit or just the gold as a negative control. First, copper TEM grids were treated with piliform, carbon-coated and glow-discharged. Samples were vortexed and fixed by mixing with a 4% PFA solution in a 1:1 ratio, for 10 min at room temperature. Each grid was left for 20 min with 10 µL of sample. The grids were then floated sequentially on drops of PBS (5 min), bovine serum albumin 1% in PBS (10 min), and then either added to PBS (for negative controls) or the primary antibody CD9 (1% in PBS) for 60 min. After washing in PBS (5 min × 3), all samples were incubated with secondary gold-conjugated antibody (1% in PBS) for 40 min. A final PBS triplicate wash was followed by fixation in 1% glutaraldehyde (5 min), deionised water (5 min) and stained in 1% uranyl acetate (5 min). The samples were imaged once dry.

### Sample preparation for mass spectrometry

Samples were prepared for mass spectrometry based on previously used methods^7,25^, using 6 technical replicates. The EVs in the samples were lysed by adding 100 µL of RIPA buffer (Sigma-Aldrich) for 10 min followed by mechanical disruption in a Bioruptor Plus (Diagenode) for 20 cycles (30 s on, 30 s off) using the low setting. 700 µL of − 20 °C acetone was added to each sample before incubation at − 20 °C overnight. The samples were then centrifuged at 18,200× *g* for 30 min at 4 °C. The supernatant was removed and 500 µL of − 20 °C 99.5% ethanol was added. The samples were centrifuged again at 18,200× *g* for 30 min at 4 °C. The supernatant was removed, and the samples were dried in a SpeedVac (miVAC DUO) at 40 °C for 10 min and resuspended in 50 µL ammonium bicarbonate (ABC) 100 mM.

The samples were prepared for mass spectrometry by proteolytically digesting the proteins using a trypsin double digestion. First, 2.3 µL of solution containing 10 M urea and 50mM ABC in LC-grade water, along with 2 µL of 0.5 µg/µL sequencing grade trypsin (Promega) were added, and the samples incubated for 30 min at 37 °C. Next, 22.7 µL of the ABC-urea solution were added and the samples incubated for 30 min at room temperature. The cysteine bonds were reduced through addition of 0.5 µL 500 mM tris(2-carboxyethyl)phosphine (TCEP), giving a final concentration of 3.23 mM TCEP, and the samples were incubated for 1 h at 37 °C. Next, 1 µL of iodoacetamide (500 mM) was added yielding a concentration of 6.37 mM and the samples were incubated for 30 min in the dark at room temperature. Following dilution with 125 µL 100 mM ABC, 2 µL of trypsin were added and the samples incubated for 16 h at 37 °C. The samples were then acidified using 10% trifluoroacetic acid (TFA) to a pH of 2–3 and the peptides purified using C18 reversed phase columns (Harvard apparatus Ultra-Micro SpinColumns Silica C18). The purified peptides were dried in a SpeedVac (miVAC DUO) and resuspended in 10 µL of 2% acetonitrile, 0.2% formic acid.

### Liquid chromatography tandem mass spectrometry (LC-MS/MS)

The peptides from each sample were measured on a Q Exactive HFX (Thermo Fisher Scientific) connected to a Dionex UltiMate 3000 RSLCnano System (Thermo Fisher Scientific) using data-independent acquisition (DIA). Peptides were separated on a µPAC Neo HPLC 50 cm column (Thermo Fisher Scientific) at 55 °C and maximum pressure of 350 bar. The peptides were eluted using a nonlinear gradient of acetonitrile in aqueous formic acid (0.1%). Starting at 3% acetonitrile and increasing to 25% in 84 min, then increasing to 38% in 11 min, and finally washing the column with 99% for 10 min before equilibrating the column with 1% acetonitrile for 10 min. A full MS scan of resolution 120,000 for a mass range of 350–1650 m/z was followed by 28 full fragmentation MS/MS scans (resolution 30,000) using isolation windows of variable size and overlap of 0.5 m/z with the previous window. Higher-energy collision-induced dissociation with normalized collision energy of 30 was used to fragment the precursor ions in the isolation windows. Automatic gain control was set to 3 × 10^6^ for MS and MS/MS.

### Mass spectrometry data analysis

Data were stored and managed using openBIS^26^. DIA-NN^27^ 1.8.1 was used for protein identification, filtered at 1% false discovery rate (FDR) and using a predicted spectral library from the reference proteomes of *Homo sapiens* (UniProt proteome ID UP000005640_9606). The retention time (RT) was calibrated using iRT peptides. Intensities were quantified from the DIA-NN report with DPKS^28^ utilizing the MaxLFQ method for quantification at protein level. The protein intensity matrix was exported and analyzed using RStudio 4.3.2. In total, 943 proteins were identified between all samples, after discarding proteins identified with only 1 peptide. Protein identification and relative abundance comparisons were made for immuno-acoustic versus acoustic, as well as immuno-acoustic versus plasma antibody. Protein interaction networks for uniquely identified proteins and significantly abundant proteins were made using STRING^29^.

## Results

### Validation of the immunoaffinity bead functionalization

10 μm silica-based seed particles have previously been used to enable rapid acoustic trapping of EVs from plasma^11^. Here we use silica-based particles of the same size, albeit with a Protein A functionalized coating which is known to bind specifically to the Fc region of polyclonal and monoclonal IgG-type antibodies. To ensure that this binding to the proteinA-silica beads works as intended, we followed the incubation and washing protocol (see Materials and Methods) by incubation with a fluorescently conjugated CD9 antibody. As shown in Figure S1, 93% of the silica beads were positive for PE, indicating that the silica beads were successfully functionalized with anti-CD9.

### Extracellular vesicle characterization

**Fig. 3:**
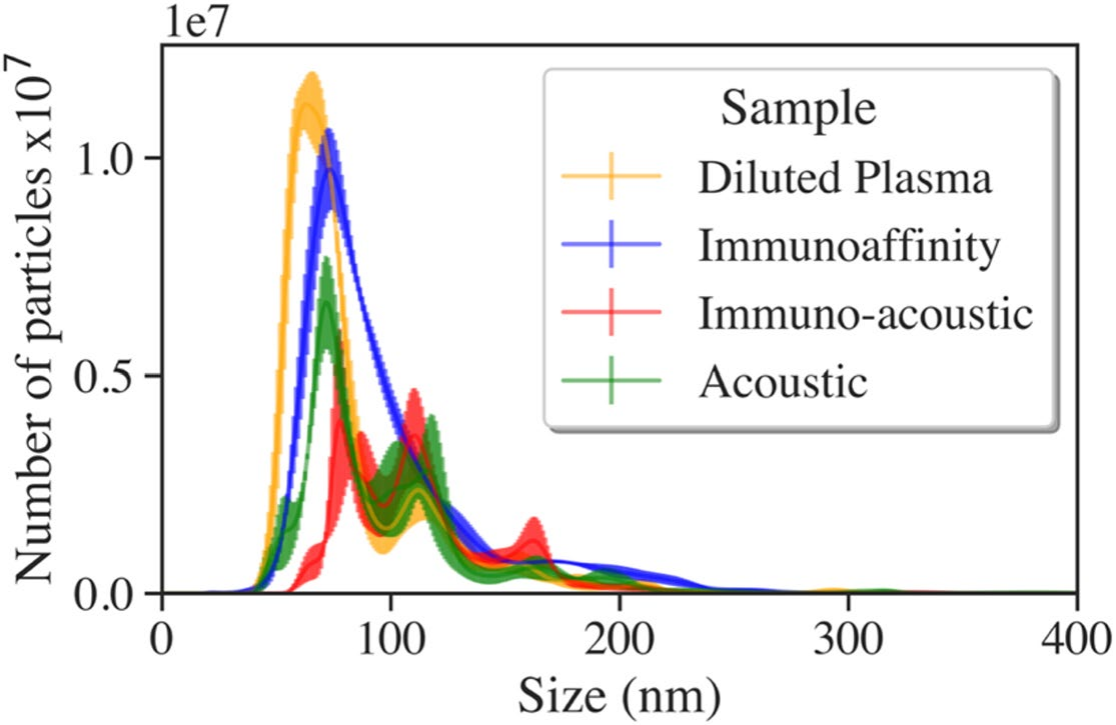
Size distribution of particles in 10 000× diluted plasma and each of the isolated EV samples, indicating ~ 90% particles below 150 nm in diameter in all samples. Number of samples for each condition (*n* = 1), number of measurements on each sample (*n* = 3). Each sample’s size distribution was calculated from > 3000 particle tracks.

The nanoparticle tracking analysis (NTA) showed a size distribution of particles in both the acoustic samples and the immuno-acoustic samples around 50–200 nm (Fig. 3), which is in the size range of EVs. The results are summarized in Table 1. Particles below 150 nm in size, indicative of small EVs, made up ~ 90% (Dv(90) < 150 nm) of all particles. The means of all size distributions lie within the standard deviations of each other. The total number of particles measured in all sample groups was above 2 × 10^8^.

**Table 1:** Size distribution data from nanoparticle tracking analysis.

Sample	Particle number ×10^8^	Particle size distribution (nm)		
		Mode ± SE	Mean ± SD	Dv(90) ± SE
Diluted plasma	4.3 ± 0.1*	63 ± 2	83 ± 36	126 ± 3
Immunoaffinity	4.7 ± 0.2	74 ± 1	98 ± 40	153 ± 3
Immuno-acoustic	2.1 ± 0.1	90 ± 8	113 ± 35	162 ± 1
Acoustic	2.8 ± 0.2	72 ± 1	100 ± 44	152 ± 7

SD is standard deviation and SE is standard error. The point Dv(90) in the size distribution is the point below which 90% of the total particle volume is contained. *Plasma was run after diluting 10,000 times.

It should be noted that the particle data in Fig. 3; Table 1 provides an overview of the size distribution of particles in the sample groups, and not directly comparative numbers of EVs, nor capture efficiency for different techniques. This is because NTA measures all particles above ~ 50 nm that scatter light, and not only EVs. Additionally, NTA measurements are semi-quantitative, as measurement accuracy is dependent on particle concentration in the sample.

**Fig. 4:**
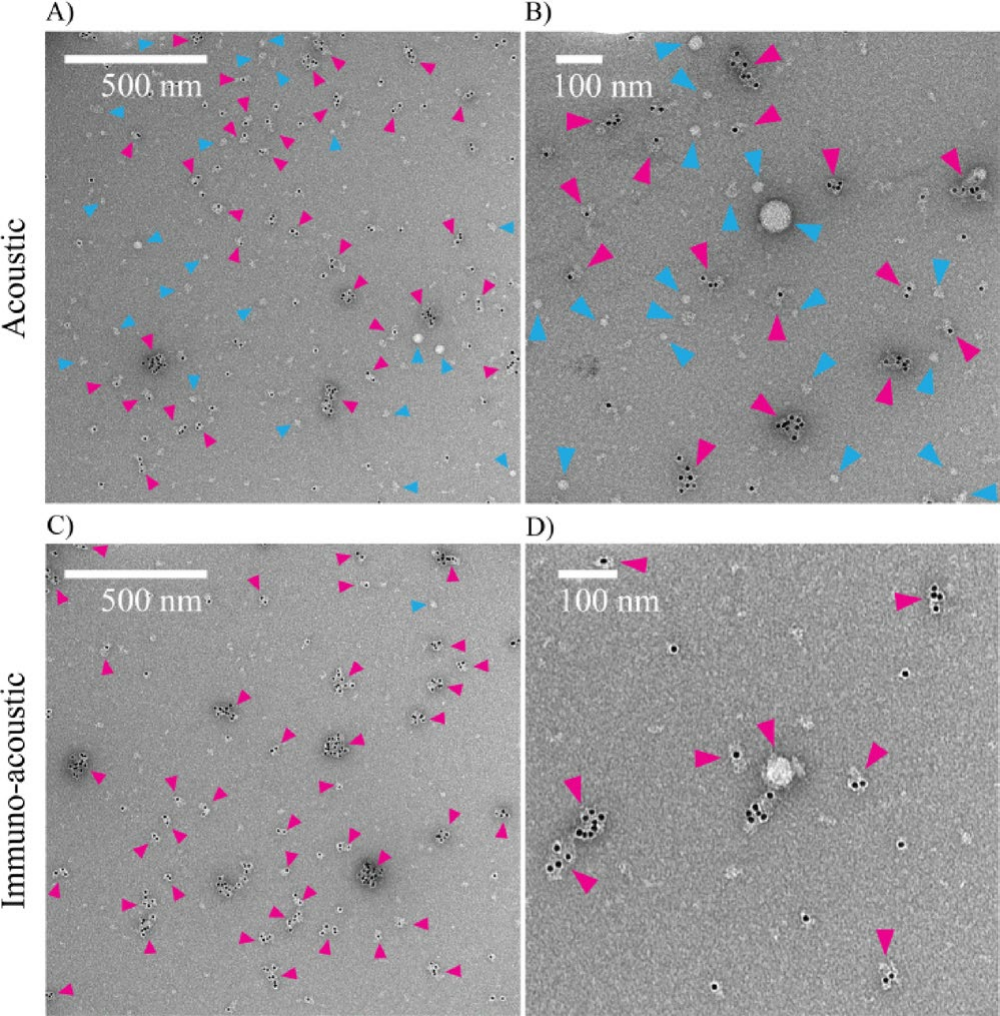
TEM images of immunogold (10 nm) labeled CD9 positive (magenta) and non-labeled (blue) extracellular vesicles in acoustic and immuno-acoustic samples, two regions are shown with different magnifications as indicated by the scale bars (*n* = 1). (**A**, **B**) Acoustically isolated samples contained both labelled EVs and non-labelled EVs. (**C**, **D**) EVs isolated immuno-acoustically were almost all labeled.

CD9 has previously been found to be highly prevalent on the membranes of small EVs, particularly in exosomes^1,2^. We investigated the CD9 expression of EVs by transmission electron microscopy (TEM) analysis. Figure 4 shows that there were many EVs present in both the acoustic and immuno-acoustic samples. TEM images of the CD9 positive EVs in raw plasma and immunoaffinity samples, along with negative staining controls can be found in Figure S2. As with the NTA, our TEM results indicated many small EVs in all samples, many of these were found to be positive for CD9 (Fig. 4). The detection limit of NTA is limited by the size and refractive index of the particles, for EVs this limit is around 50 nm which led to the modal size of 70–90 nm. As we^10^ and others^30^ have previously reported and seen here (Fig. 4), TEM detects many smaller EVs that are not detectable in NTA. This may be due to TEM having a lower limit of detection, dehydration shrinkage in the TEM preparation and the fact that NTA overestimates size by measuring the hydrodynamic radius. Despite being a size-dependent isolation method, acoustic trapping has been previously found to be more effective at isolating small EVs (mostly < 150 nm) from plasma than ultracentrifugation (mostly > 150 nm EVs were isolated)^9^. Figure 4 presents images of EVs from an acoustic sample, with EVs both positive and negative for CD9. This was not surprising, as there are known to be a large number of EVs in plasma and the immunoaffinity beads are unlikely to deplete all EVs containing CD9 from the plasma. Furthermore, it is possible that some of the immunoaffinity isolated EVs could have become detached from the seed particles during the fractionation step. The immuno-acoustic sample showed very few unlabeled EVs, indicating that a subpopulation of CD9^+^ EVs has been isolated (Fig. 4).

In summary, the results from NTA and TEM analysis show that immuno-functionalized silica seed particles were successfully used in the acoustic trap to isolate EVs from plasma and subsequently generated one purified fraction of CD9^+^ EVs and one fraction of acoustically isolated EVs.

### Proteomic analysis of isolated EV fractions

**Fig. 5:**
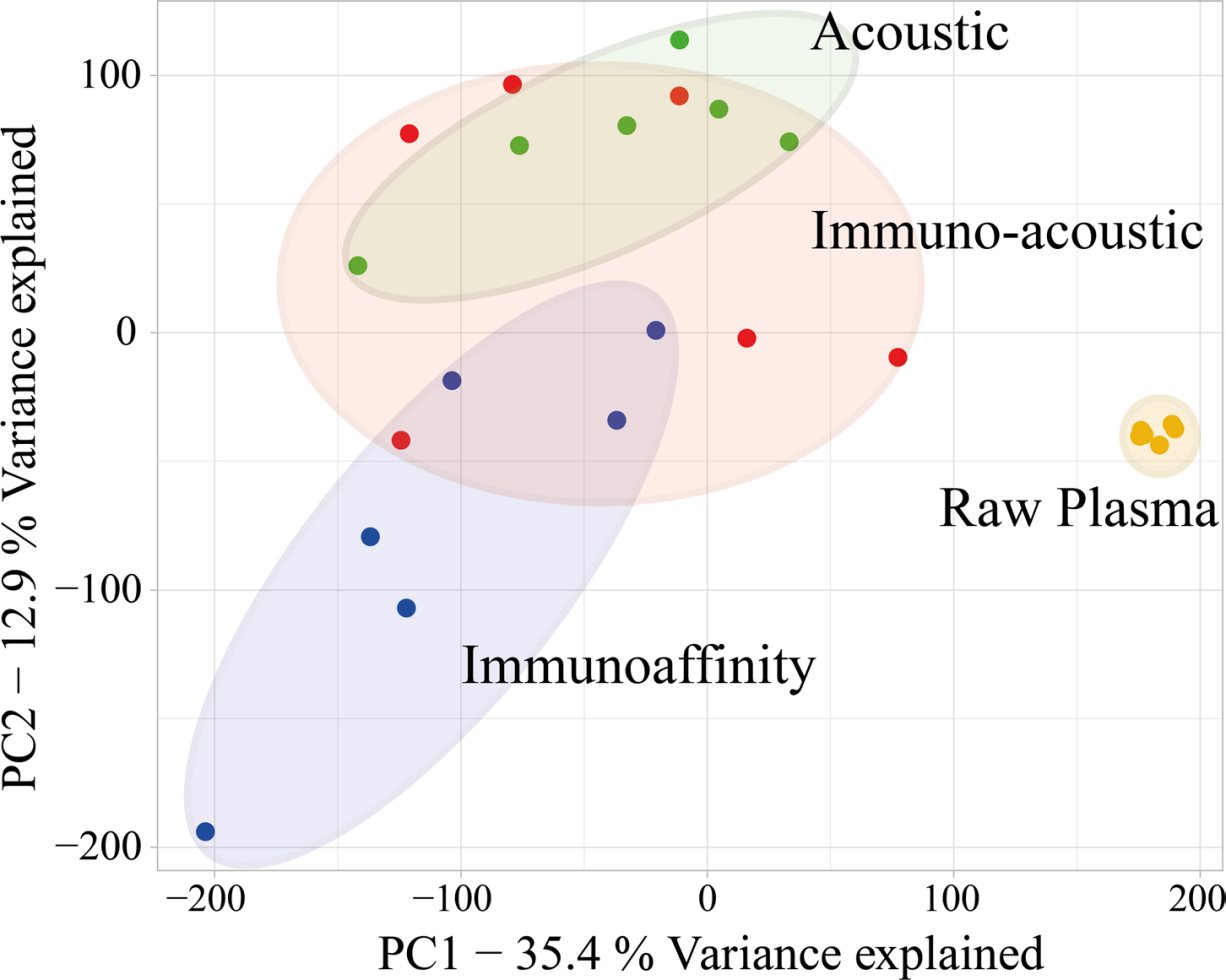
PCA plot of all samples (*n* = 6 for each sample group). The largest differences are between samples with isolated EVs and plasma (yellow). The isolated EVs are as indicated: acoustic (green), immuno-acoustic (red) and immunoaffinity (blue).

As expected, the protein content of the three groups of samples where EVs were enriched from plasma—by immunoaffinity, acoustic or immuno-acoustic techniques—were distinct from the raw plasma samples. This was made apparent by principal component analysis (PCA) of all protein intensities of all samples (Fig. 5) and by unsupervised clustering (presented as a heatmap in Figure S3). This reinforces the value of enriching EVs from biofluids, washing off background proteins.

**Fig. 6:**
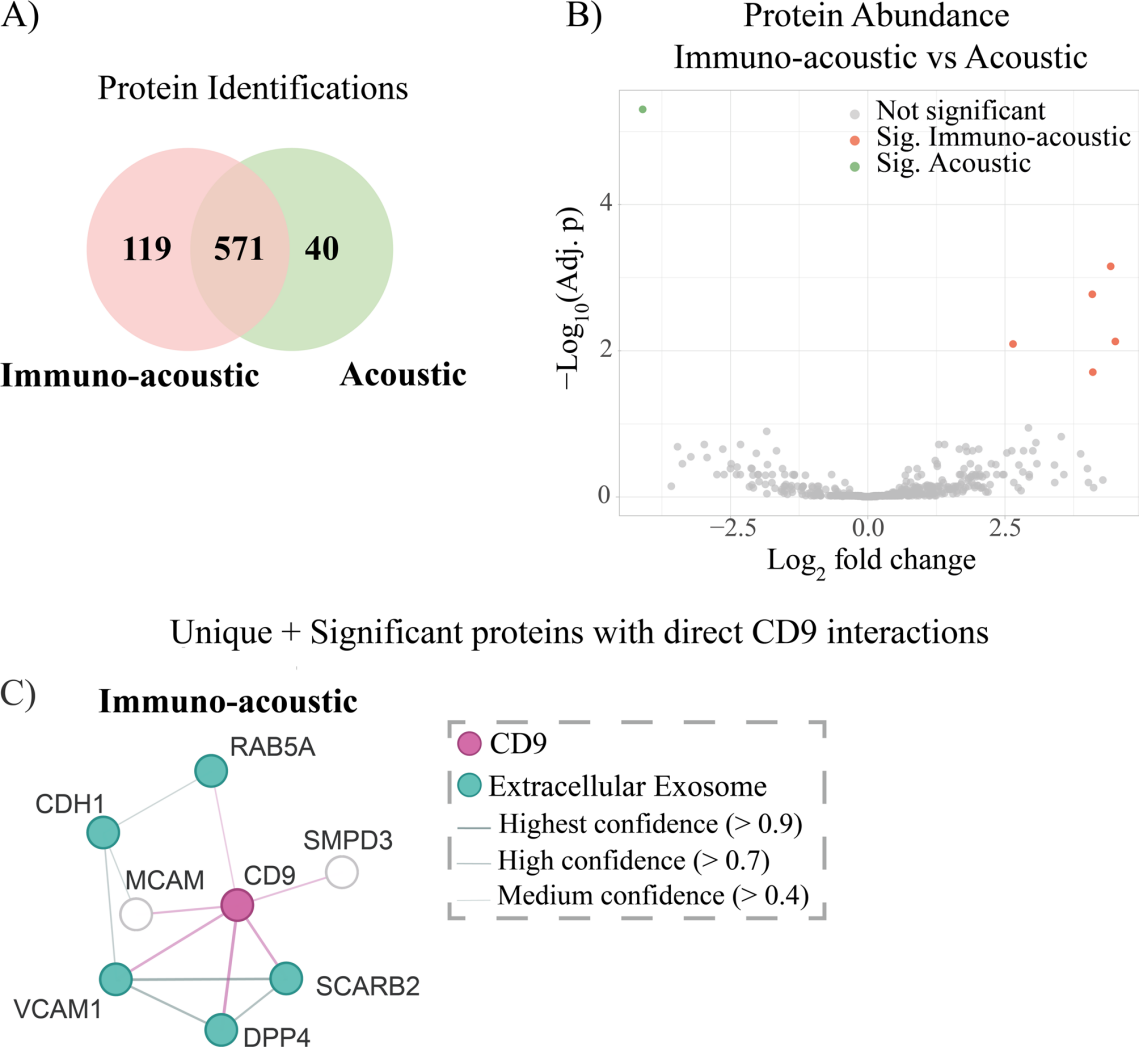
(**A**) Venn diagram of protein identifications for immuno-acoustic and acoustic samples (*n* = 6 for each sample group). Proteins were counted towards a group if it was identified in at least two samples within the group. (**B**) Differential protein abundance analysis of commonly identified proteins. Significance cut-off was for adjusted *p*-values (Benjamini-Hochberg) less than 0.05. (**C**) Protein interaction network of all proteins directly interacting with CD9 that are significant and unique in immuno-acoustic, compared to acoustic samples. The network was generated with STRING and the thickness of the edges indicates the confidence of the interaction. Proteins labeled in teal are associated with gene ontology GO:0070062 Extracellular Exosome. CD9 (labeled in magenta) has been artificially added to the network.

When comparing proteins identified in the immuno-acoustic to the acoustic group, 119 unique proteins were found in the immuno-acoustic group, 40 unique proteins were found in the acoustic group and 571 proteins were found in both groups (Fig. 6A). Among the 571 proteins found in common between the groups, differential protein abundance analysis identified 5 proteins significantly abundant in immuno-acoustic samples and 1 protein significantly abundant in acoustic samples (Fig. 6B). Proteins were considered significant if the adjusted *p*-value (Benjamini-Hochberg) was less than 0.05. In total, there were 124 uniquely identified or significantly abundant proteins in the immuno-acoustic samples, and 41 uniquely identified or significantly abundant proteins in the acoustic samples.

The proteomics data shows that there is a large overlap in the protein content of the acoustically trapped EVs and the immuno-acoustic captured EVs. This is not surprising, as EV protein content has previously shown weak correlation with surface markers^2^. Additionally, the presence of CD9 positive EVs in the acoustically isolated fraction is expected and confirmed by TEM, as we do not expect the functionalized seed particles to deplete all CD9^+^ EVs from plasma. This further increases the proteomic overlap between the sample groups. Still, the fact that 119 proteins were uniquely identified in the immuno-acoustic fraction and 40 proteins were uniquely identified in the acoustic fraction, demonstrated that fractionating the EV population does to some extent give access to different proteomes.

Despite confirming the presence of CD9^+^ EVs in all sample groups by TEM, CD9 was not detected through mass spectrometry analysis. This is not surprising, as CD9 is a tightly bound membrane protein passing through the membrane four times, and previous studies have shown difficulty identifying CD9 by means of mass spectrometry^31^. Membrane proteins are notoriously challenging to access, digest and solubilize^32^. In order to assess how related the proteins in the two EV fractions were to CD9, and therefore infer the presence of CD9, uniquely identified proteins along with significantly abundant proteins within the immuno-acoustic and acoustic samples were subject to functional network analysis using STRING^29^ (Fig. 6C and S4). CD9 has been artificially added (in magenta) to each protein list to highlight interactions with CD9. For immuno-acoustic samples compared with acoustic samples, 60% of the unique or significant proteins were associated with gene ontology GO:0070062 Extracellular Exosome. For acoustic samples, this figure was 52.6%. The immuno-acoustic group identified 7 proteins with direct interactions with CD9. None of the proteins significant or unique to the acoustic samples had known interactions with CD9.

The unique and significant protein groups for immuno-acoustic and acoustic isolation both contained a high proportion of proteins associated with GO:0070062 Extracellular Exosome. This suggests that the main part of the difference is due to changes in the exosome related proteins, which further suggests that the fractionation of EVs gives access to different EV proteomes. The protein interaction networks illustrated in Figure S4 reveal that, despite the absence of CD9 detection, the unique and significant proteins identified in the immuno-acoustic group, Fig. 6C, exhibited several direct interactions with CD9. This suggests that isolating EVs using a specific surface marker allows us to access proteins that closely interact with that marker.

Accessing over 100 potentially interesting proteins unique to immuno-acoustically isolated EVs with CD9, an already abundant EV marker, is very promising. It suggests that applying this technique with a rarer cell-type specific EV marker could reveal, for example, low abundant disease-associated proteins. This would be valuable for researchers probing the role in EVs in a specific disease mechanism. Coupling this with acoustic isolation is clearly valuable not just due to the convenience of automated handling, but also because the acoustic sub-population could provide a reference for deeper differential proteomics and biomarker discovery.

**Fig. 7:**
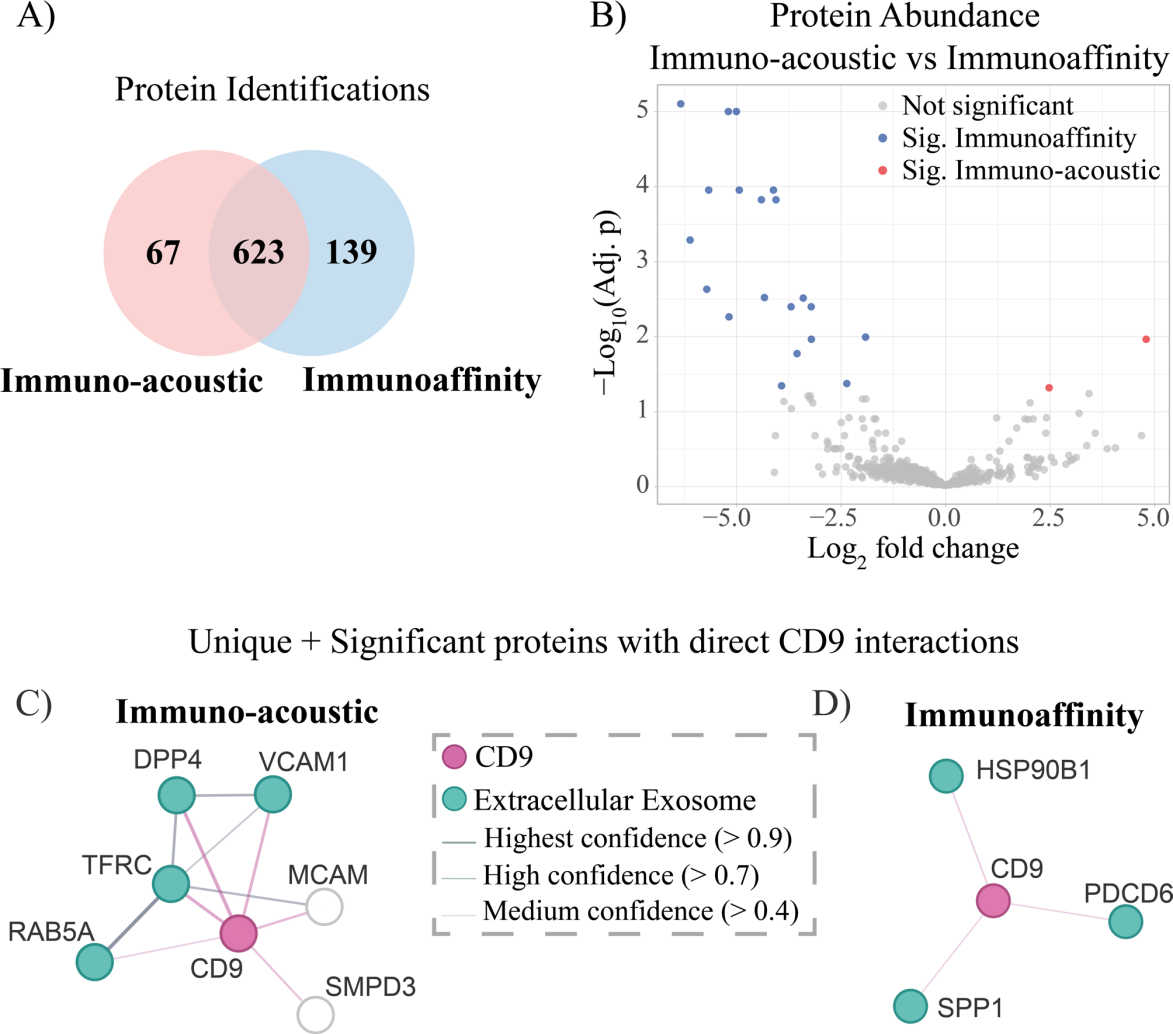
(**A**) Venn diagram of protein identifications for immuno-acoustic and immunoaffinity samples (*n* = 6 for each sample group). Proteins were counted towards a group if it was identified in at least two samples within the group. (**B**) Differential protein abundance analysis of commonly identified proteins. Significance cut-off was for adjusted *p*-values (Benjamini-Hochberg) less than 0.05. Protein interaction networks of all proteins directly interacting with CD9 significant and unique: (**C**) in immuno-acoustic compared with immunoaffinity samples, and (**D**) in immunoaffinity compared with immuno-acoustic samples. Protein interaction networks generated with STRING. The thickness of the edges indicates the confidence of the interaction. Proteins labeled in teal are associated with gene ontology GO:0070062 Extracellular Exosome. CD9 (labeled in magenta) has been artificially added to each network.

To understand how the results from our coupled technique differ from a more established method, we investigated differences in protein content between immuno-acoustically isolated and immunoaffinity isolated EVs. Across both sample groups, 829 proteins were identified with at least 2 peptides (Fig. 7A). Proteins were counted as identified within a sample group if they were found in at least two samples in the group. In total, 139 proteins were found uniquely in the immunoaffinity group, and 67 proteins were found uniquely in the immuno-acoustic group. Differential protein abundance analysis of the 623 proteins found in common between the groups revealed 20 proteins significantly enriched in the immunoaffinity group, and 2 proteins significantly enriched in the immuno-acoustic group (Fig. 7B). Proteins were counted as significantly differentially abundant if the adjusted *p*-value (Benjamini Hochberg) was lower than 0.05.

To benchmark the insight offered by the antibody-based isolation on the acoustic trapping platform, we also compared the immuno-acoustic samples with the standard immunoaffinity isolation in terms of functional network analyses of the uniquely identified and significant proteins for these groups, Figure S5. Again, CD9 was not detected for either standard immunoaffinity or immuno-acoustic based isolation. Analogous to the above comparison in Fig. 7C,D, CD9 was added artificially (in magenta) to each network, to highlight its interactions with the differentially abundant proteins. For immuno-acoustic samples compared with immunoaffinity, 59% of the unique or significant proteins were associated with gene ontology GO:0070062 Extracellular Exosome. For the reverse comparison, for standard immunoaffinity samples, this figure was 47%. When analysing the unique and significantly abundant proteins between the immuno-acoustic group and immunoaffinity group, a greater number of proteins were found to directly interact with CD9 in the immuno-acoustic group. Specifically, 6 directly interacting proteins were found for the immuno-acoustic group and 3 were found for the immunoaffinity group. Additionally, the immunoaffinity group had lower confidence in the protein interactions with CD9. Notably, this was despite the fact that a greater number of proteins overall were found either unique or significantly abundant in the immunoaffinity group, compared to the immuno-acoustic group (159 vs. 69 proteins). Together, this indicates that immuno-acoustic isolation provided better access to a purer fraction of CD9^+^ EVs than immunoaffinity based isolation in our study. Coupled with the faster processing time (8 min vs. 90 min incubation used in this study), automated handling, and simultaneous access to an additional fraction of acoustically isolated EVs, immuno-acoustic isolation displayed several advantages over immunoaffinity isolation.

## Discussion

Immunoaffinity-based isolation of EVs has previously been realized in several microfluidics devices^3,23,33−35^. These devices tend to have slow processing times, require extensive pre-purification steps and often do not demonstrate release of the immunoaffinity-isolated EVs from the functionalized beads/surfaces. Integrating immunoaffinity-based isolation with acoustic trapping allows for specificity while retaining the benefits of an acoustic trap, such as short processing times, low volume requirements, gentle forces, automated processing and a readily reusable system. In this study, we demonstrated the feasibility of immuno-acoustic trapping to process and isolate fractions of EVs, without need for extensive sample pre-processing. Downstream MS-based proteomics showed differences in the protein content within the two EV fractions obtained by the immuno-acoustic trapping technique; highlighting the additional information gained through epitope specificity during acoustic EV-isolation, compared to acoustic EV trapping alone. The combination of immunoaffinity based isolation of EVs and acoustic retention of particles has been explored previously, by using two microfluidic modules in series: one for incubating functionalized microparticles with EVs and one for the acoustic collection of the microparticles^22^. This means that the acoustic forces were only used to separate microparticles from their surrounding fluid and washing unbound species, in principle analogous to the standard microbead immunoaffinity based EV isolation used as reference in the study above. The methodology presented in our study displayed several advantages over this approach. First, taking advantage of the acoustic seed particle trapping of nanoparticles enabled us to simultaneously capture two EV fractions from a biological sample. Second, we demonstrated EV isolation directly from plasma samples, as compared to already purified EVs. Third, the processing time was significantly faster (8 min compared to ~ 1 h 40 min). It should however be noted that the chosen immunoaffinity protocol may not be optimal and faster protocols achieving equivalent results could potentially be implemented.

The faster processing times could be explained by enrichment of EVs in vicinity of the functionalized seed particles, increasing the interaction between CD9^+^ EVs and the antibody. Since in the microfluidic approach the plasma is continuously passed over the seed particle cluster, more plasma comes into contact with the functionalized surface. This may be further aided by streaming vortices generated in the acoustic field. Additionally, EVs in the sample will experience acoustic radiation forces attracting them to the seed particle cluster, further increasing the contact between the functionalized seed particles and CD9^+^ EVs.

One limitation with the acoustic technique is that currently samples cannot be processed in parallel without multiple instruments, this means that scaling up to 80 samples (~ 1.4 mL plasma with the parameters used in this study) would have a total processing time of nearly 11 h, for example. Our technique addresses the need for methods which can get more information from a cohort of distinct, small patient samples, rather than tackling one large, pooled sample. This makes it useful for applications within research and diagnostics, but not for purifying several litres of EVs produced for therapeutic purposes.

Immuno-acoustic isolation provided a purer isolation than immunoaffinity isolation in our study. One explanation for this is that the washing mechanism is different in the acoustic trap, with continuous flow of clean buffer over the acoustically retained EVs. This is different compared to the washing protocol for immunoaffinity, where the particles are pelleted using centrifugation, the supernatant removed and the particles resuspended in clean buffer. It is currently unknown to which extent this affects the efficiency of the washing procedure but previous studies have demonstrated significant reduction of background proteins in samples where EVs have been isolated by acoustic trapping^7,8^. Another factor could be IgGs from the plasma sample binding to protein A, which would reduce EV recovery and could have occurred to a different extent for the two immuno-isolation methods. However, rabbit IgG (for the anti-CD9) is known to bind very strongly to protein A, so human IgGs are not expected to outcompete excessively.

A key aspect of the method presented in this work is that two EV populations are simultaneously isolated from the same sample. Firstly, this increased total protein coverage (indicated by the uniquely identified proteins in the immuno-acoustic fraction). Secondly, more nuanced information was obtained in that it was possible to differentiate between proteins enriched in the more general EV population (acoustic fraction) compared with the specific subpopulation (immuno-acoustic fraction). In this case the subpopulation is CD9^+^ EVs, however the use of a cell-specific or rarer surface marker could lead to even greater differential expression and more clinically relevant information about the function of an EV subpopulation. We used a flexible method to enable the antibody to be changed without buying new beads, however future studies could investigate whether a covalently bonded antibody would result in higher recovery.

It should be noted that the acoustically isolated fraction will in most cases contain EVs that are positive for the targeted surface marker, as the functionalized beads are unlikely to deplete all targeted EVs from the supernatant. It is also possible that EVs bound to the antibody release during the fractionation step and would then end up in the acoustically isolated fraction. This should be kept in mind if downstream analyses comparing the two EV populations are carried out. However, these effects do not affect the purity of the EVs in the immuno-acoustically isolated fraction.

## Conclusions

For the first time, we have coupled antibody-based capture with acoustic trapping for isolation of EVs from blood plasma samples. Our methodology enables simultaneous and rapid isolation of two EV fractions: one general plasma EV population acoustically trapped, and a second subpopulation of EVs positive for the target membrane protein, in this case CD9. The isolated EVs can be fractionated to produce subpopulations of plasma EVs and EVs positive for a target membrane protein. Following NTA, TEM and downstream proteomics, we confirmed the presence of CD9^+^ EVs and the enrichment of CD9 associated proteins. Despite the difficulty in identifying CD9 by mass spectrometry, we show that it is possible to uncover biological information relating CD9^+^ EVs. By implementing antibody-based specific isolation into an acoustofluidic platform we can capitalize on small volume requirements, short processing times, as well as automated enrichment and washing which combine to make this methodology attractive for handling processing biobank samples. Furthermore, we present a flexible methodology which could be applied to target any (or multiple) antigen(s) of interest in any cell-free biofluid containing EVs. Since two subpopulations are fractionated with this immuno-acoustic isolation method, it offers novel opportunities for differential proteomics between rarely compared EV populations.

## Supplementary Information

Below is the link to the electronic supplementary material.

Supplementary Material 1

## Data Availability

The Supporting Information is available free of charge online in the file SupportingInformation.pdf, Figures S1–S5. These provide additional results including flow cytometry of fluorescently functionalized silica beads, TEM of all sample types, protein heatmap for all samples and extensive protein interaction networks. The datasets generated and analysed during the current study are available in the MassIVE repository [doi:10.25345/C5JW8709Q], ftp://massive-ftp.ucsd.edu/v10/MSV000098181/.

## Abbreviations

DIA: Data independent analysis
EV: Extracellular vesicle
FACS: Fluorescence activated cell sorting
MS: Mass spectrometry
NTA: Nanoparticle tracking analysis
TEM: Transmission electron microscopy
