# Supplementary material for: Immuno-acoustic trapping for extracellular vesicle subpopulations
Source: Sci Rep. 2025 Dec 31;15:45805. doi: 10.1038/s41598-025-33842-6 (PMC12756313; doi:10.1038/s41598-025-33842-6)
Supplement: Supplementary file 1 — Supplementary Material 1 [file 41598_2025_33842_MOESM1_ESM.pdf]

Supporting Information

Immuno-acoustic trapping for extracellular vesicle subpopulations

Axel Broman [a†], Megan Havers [a*†], Roman Sattarov [b] and Thomas Laurell [a]*

[a]Department of Biomedical Engineering, Lund University, Lund, Sweden,

[b]Department of Clinical Sciences, Lund University, Lund, Sweden

* Corresponding authors axel.broman@bme.lth.se, megan.havers@bme.lth.se

† These authors contributed equally

Supplementary Figures

Figure S1: Flow cytometry of functionalized silica seed particles

Figure S2: Transmission electron microscopy images of immunogold labelled extracellular vesicles, and negative controls.

Figure S3: Protein intensity heatmap of all proteins detected across all samples

Figure S4: Protein interaction network of unique and significant proteins comparing immuno-acoustic isolation to acoustic isolation

Figure S5: Protein interaction network of unique and significant proteins comparing immuno-acoustic isolation to immunoaffinity isolation

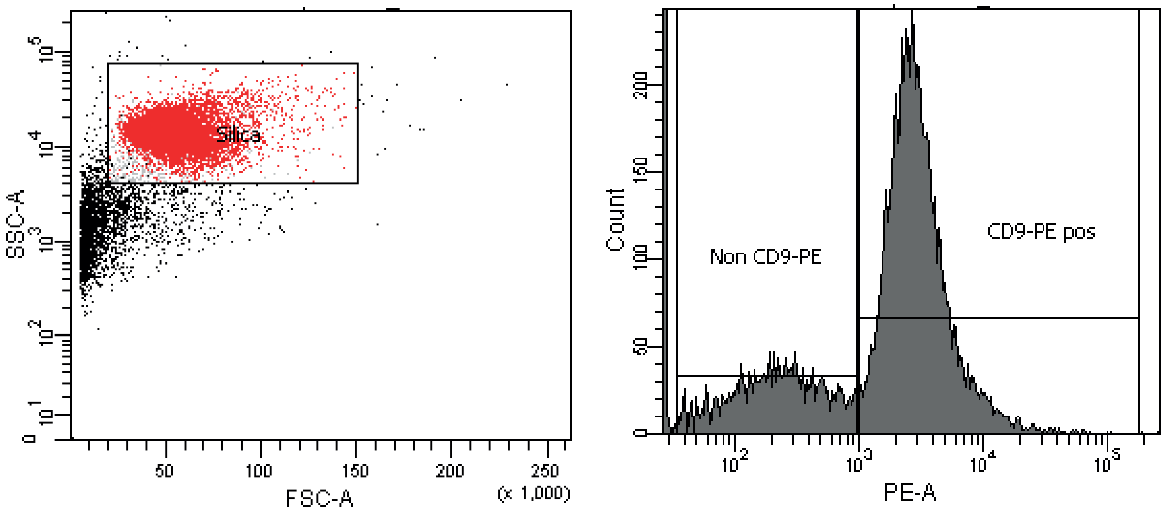

Tube: pos cntr_001

Population	#Events	%Parent	%Total
All Events	10,000	####	100.0
Silica	7,371	73.7	73.7
Non CD9-PE	485	6.6	4.9
CD9-PE pos	6,855	93.0	68.6

Figure S1: Flow cytometry plots showing 93% positive labelling of silica seed particles with fluorescent CD9-PE. Side scatter and forward scatter used to gate for silica seed particles (~10 µm), followed by PE intensity gating based on unlabeled silica seed particles (0.3% in the unlabeled silica reference).

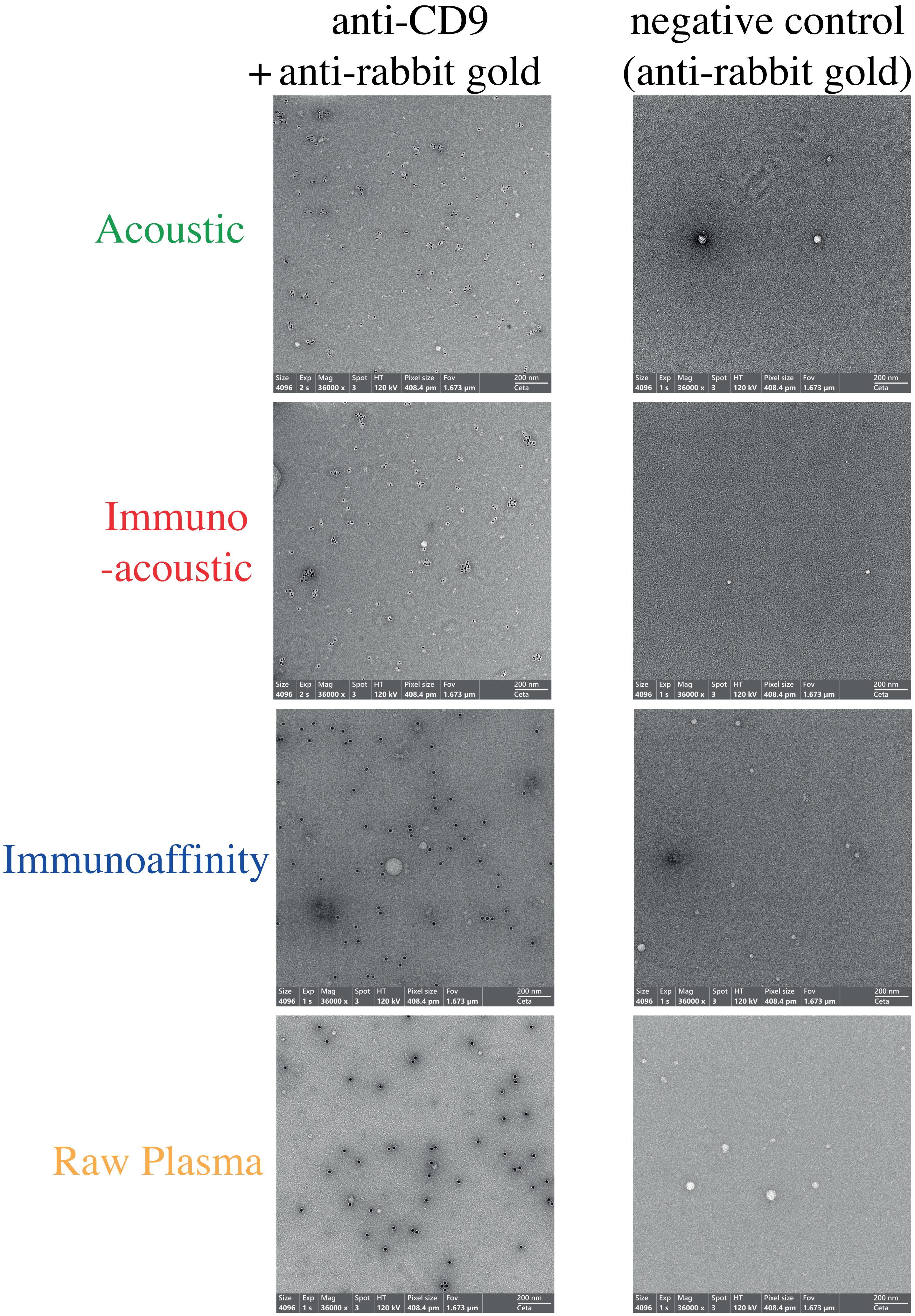

Figure S2: Transmission electron microscopy images of EVs from the different sample groups, stained CD9^{+} EVs and negative control staining showing no gold nanoparticles. The anti-rabbit gold were 10 nm in acoustic and immuno-acoustic samples, and 15 nm in immunoaffinity and raw plasma samples.

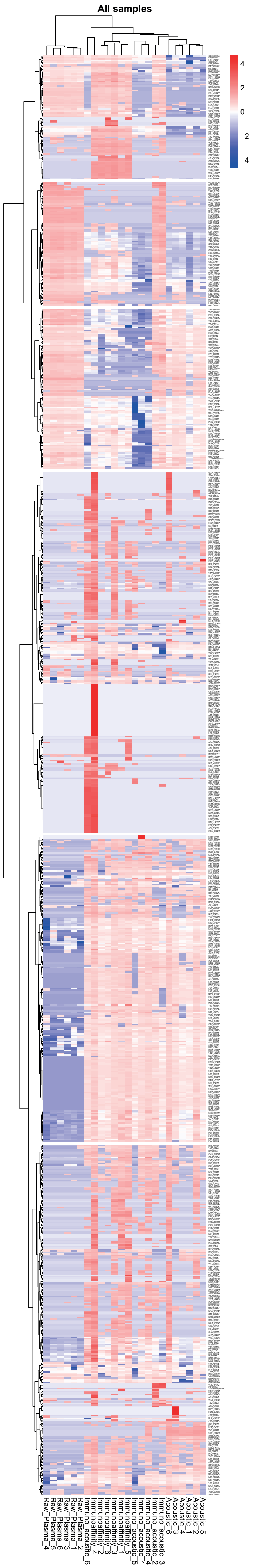

Figure S3: Protein intensity heatmap of all proteins found in all samples. Missing values have been assigned an intensityof 0. The intensities have been log2 transformed and row-normalized. The heatmap is clustered with unsupervised clustering and the legend gives the z-score.

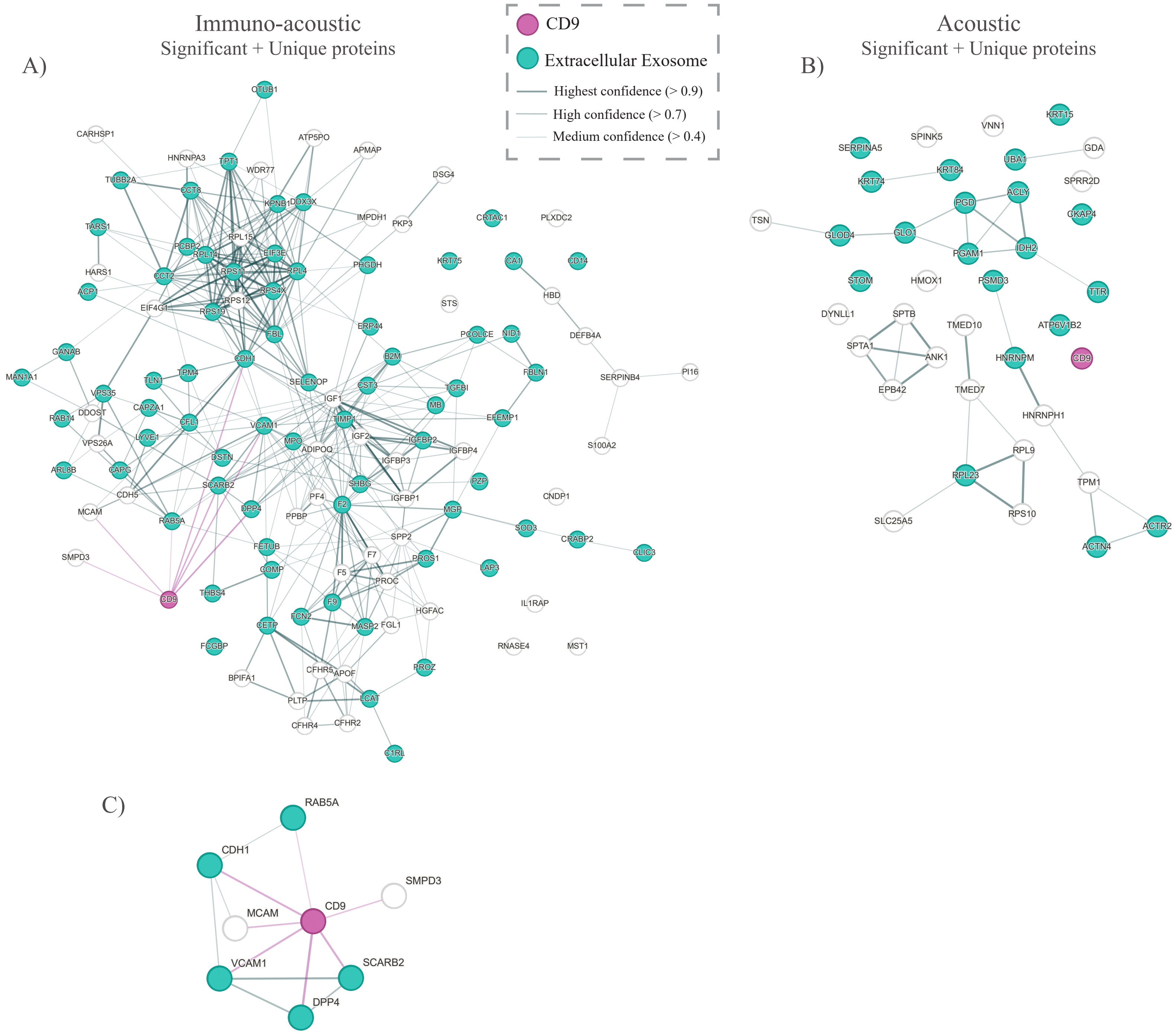

Figure S4: Protein interaction networks generated with STRING. The thickness of the edges indicates the confidence of the interaction. Proteins labeled in teal are associated with gene ontology GO:0070062 Extracellular Exosome. CD9 (labeled in magenta) has been artificially added to each network. A) Proteins uniquely identified or significantly abundant in immuno-acoustic samples, as compared with acoustic samples. There are 7 proteins that directly interact with CD9. B) Proteins uniquely identified or significantly abundant in acoustic samples, as compared with immuno-acoustic samples. There are no interactions with CD9. C) Protein interaction network of all proteins directly interacting with CD9 from A).

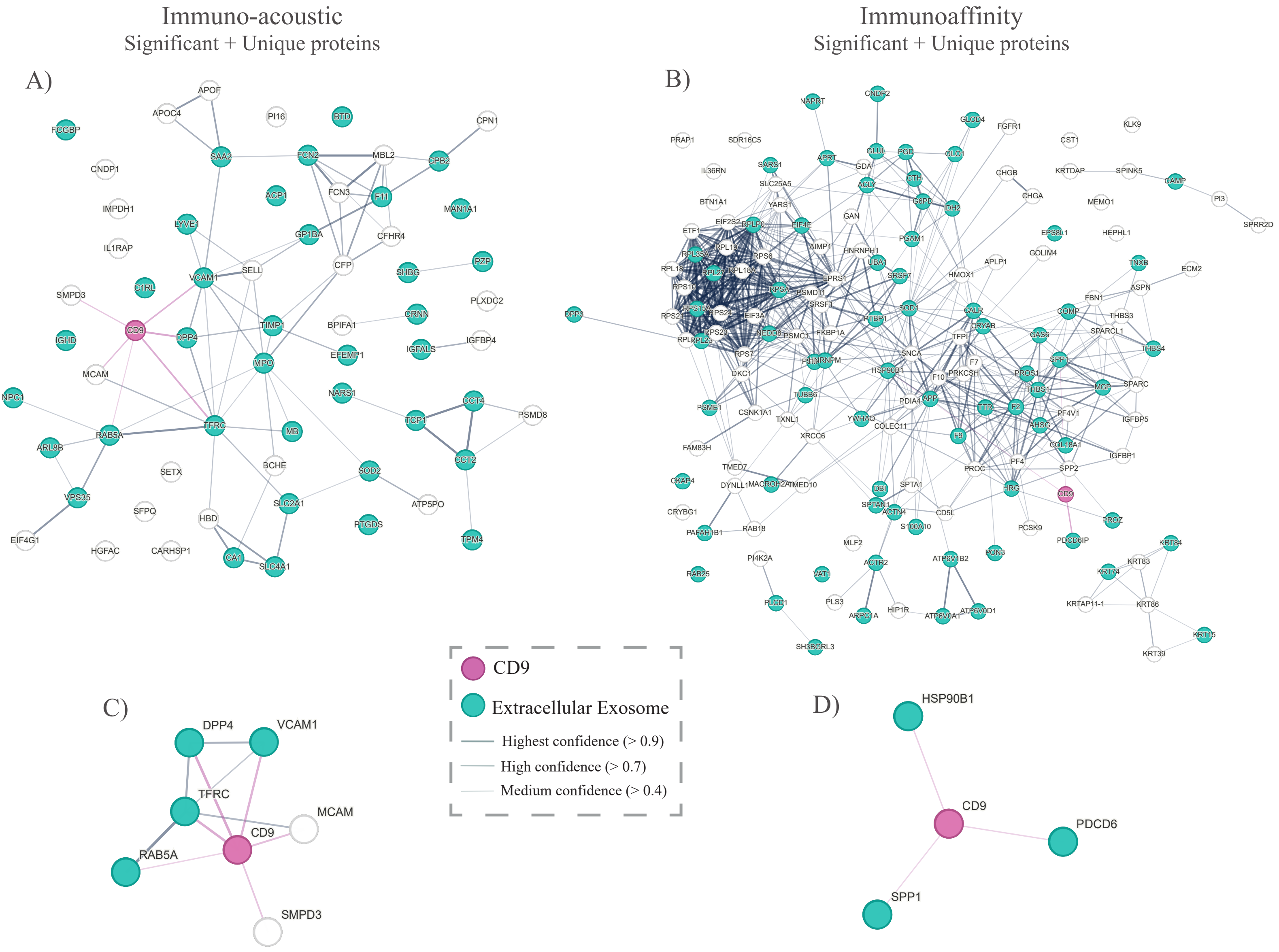

Figure S5: Protein interaction networks generated with STRING. The thickness of the edges indicates the confidence of the interaction. Proteins labeled in teal are associated with gene ontology GO:0070062 Extracellular Exosome. CD9 (labeled in magenta) has been artificially added to each network. A) Proteins uniquely identified or significantly abundant in immuno-acoustic samples, as compared with immunoaffinity samples. There are 6 proteins that directly interact with CD9. B) Proteins uniquely identified or significantly abundant in immunoaffinity samples, as compared with immuno-acoustic samples. There are 3 proteins directly interacting with CD9. C) Protein interaction network of all proteins directly interacting with CD9 from A). D) Protein interaction network of all proteins directly interacting with CD9 from B).
